# Saturated fatty acid– and/or monounsaturated fatty acid–containing phosphatidic acids selectively interact with heat shock protein 27

**DOI:** 10.1016/j.jbc.2023.103019

**Published:** 2023-02-13

**Authors:** Naoto Yachida, Fumi Hoshino, Chiaki Murakami, Masayuki Ebina, Yuri Miura, Fumio Sakane

**Affiliations:** 1 Department of Chemistry, Graduate School of Science, Chiba University, 1-33 Yayoi-cho, Inage-ku, Chiba, Japan; 2 Institute for Advanced Academic Research, Chiba University, 1-33 Yayoi-cho, Inage-ku, Chiba, Japan; 3 Research Team for Mechanism of Aging, Tokyo Metropolitan Institute of Gerontology, Itabashi, Tokyo, Japan

**Keywords:** phosphatidic acid, heat shock protein 27, diacylglycerol kinase, cancer, saturated fatty acid, BN-PAGE, Blue native polyacrylamide gel electrophoresis, Chol, cholesterol, CL, cardiolipin, DG, diacylglycerol, DGK, diacylglycerol kinase, ERK, extracellular signal-regulated kinase, HSP, heat shock protein, *K*_d_, dissociation constant, LC-MS/MS, liquid chromatography-tandem mass spectrometry, MAPK, mitogen-activated protein kinase, MUFA, monounsaturated fatty acid, PA, phosphatidic acid, PABP, phosphatidic acid-binding protein, PC, phosphatidylcholine, PDE, cAMP phosphodiesterase, PI, phosphatidylinositol, PI(4)P, phosphatidylinositol-4-monophosphate, PI(4,5)P_2_, phosphatidylinositol-4,5-bisphosphate, PKC, protein kinase C, PLD, phospholipase D, PS, phosphatidylserine, PUFA, polyunsaturated fatty acid, SFA, saturated fatty acid

## Abstract

Diacylglycerol kinase (DGK) α, which is a key enzyme in the progression of cancer and, in contrast, in T-cell activity attenuation, preferentially produces saturated fatty acid (SFA)– and/or monounsaturated fatty acid (MUFA)–containing phosphatidic acids (PAs), such as 16:0/16:0-, 16:0/18:0-, and 16:1/16:1-PA, in melanoma cells. In the present study, we searched for the target proteins of 16:0/16:0-PA in melanoma cells and identified heat shock protein (HSP) 27, which acts as a molecular chaperone and contributes to cancer progression. HSP27 more strongly interacted with PA than other phospholipids, including phosphatidylcholine, phosphatidylserine, phosphatidylglycerol, cardiolipin, phosphatidylinositol, phosphatidylinositol 4-monophosphate, and phosphatidylinositol 4,5-bisphosphate. Moreover, HSP27 is more preferentially bound to SFA- and/or MUFA-containing PAs, including 16:0/16:0- and 16:0/18:1-PAs, than PUFA-containing PAs, including 18:0/20:4- and 18:0/22:6-PA. Furthermore, HSP27 and constitutively active DGKα expressed in COS-7 cells colocalized in a DGK activity–dependent manner. Notably, 16:0/16:0-PA, but not phosphatidylcholine or 16:0/16:0-phosphatidylserine, induced oligomer dissociation of HSP27, which enhances its chaperone activity. Intriguingly, HSP27 protein was barely detectable in Jurkat T cells, while the protein band was intensely detected in AKI melanoma cells. Taken together, these results strongly suggest that SFA- and/or MUFA-containing PAs produced by DGKα selectively target HSP27 and regulate its cancer-progressive function in melanoma cells but not in T cells.

Diacylglycerol kinase (DGK) phosphorylates diacylglycerol (DG) to convert to phosphatidic acid (PA) (1, 2, 3, 4) and participates in a great variety of pathological and physiological functions (5, 6). Mammalian DGK consists of ten isoforms, which are divided into five groups (types I–V) (1, 2, 3, 4). Type I DGK consists of the α, β, and γ isozymes. DG-binding proteins are C1 domain-containing proteins, including conventional protein kinase C (PKC), novel PKC, and Ras guanine nucleotide-releasing protein (7, 8, 9, 10). There are many PA-binding proteins (PABPs) (more than 70), such as atypical PKC (PKCζ), novel PKC (PKCδ and ε), C-Raf, cAMP phosphodiesterase (PDE) 4A1, Opi1p, sporulation-specific protein 20p, α-synuclein, creatine kinase muscle type, L-lactate dehydrogenase A, Praja-1, and synaptojanin-1 (11, 12, 13, 14, 15). Moreover, the number of PABPs is still growing.

DGKα (16, 17) has tandem Ca^2+^-binding EF-hand motifs and is activated by Ca^2+^ (16, 18, 19, 20, 21, 22). DGKα is abundantly expressed in T lymphocytes/thymus (16) and cancer cells, including melanoma (23), hepatocellular carcinoma (24) and mesenchymal glioblastoma (25). DGKα facilitates the immune nonresponsive (nonproliferation) state known as anergy in T lymphocytes (26, 27, 28). In contrast to T cells, DGKα attenuates apoptosis and promotes the proliferation of melanoma (23, 29, 30) and hepatocellular carcinoma cells (24) and enhances epithelial–mesenchymal transition (metastasis) of glioblastoma (25). In addition, DGKα has been reported to activate angiogenesis signaling (31). Therefore, the inhibition of DGKα activity may suppress cancer progression (32, 33, 34). As expected, DGKα-selective inhibitors and DGKα-specific siRNA caused apoptosis and inhibited the proliferation and epithelial–mesenchymal transition of several cancer cell lines (23, 24, 25, 30, 32). Therefore, DGKα has reverse roles in T cells (attenuator) and cancer cells (enhancer) (6, 34). It remains unclear how DGKα differently functions in these cells.

We recently found that DGKα preferentially generated saturated fatty acid (SFA)- and/or monounsaturated fatty acid (MUFA)-containing PAs, such as 16:0/16:0-, 16:0/18:0-, and 16:0/16:1-PA in serum-starved AKI melanoma cells (30). Moreover, DGKα was indicated to produce broad SFA-, MUFA-, and/or polyunsaturated fatty acid (PUFA)-containing PA species, such as 14:1/16:1-, 14:0/16:1-, 14:0/16:0-, 16:1/16:2-, 16:1/16:1-, 16:0/16:1-, 16:0/16:0-, 16:0/18:1-, and 16:0/18:0-PA, in serum-starved Jurkat T cells (35). PA species generated by DGKα in melanoma and T cells overlap each other. However, unsaturation levels of fatty acids of PA species in T cells are moderately higher than those in melanoma cells. These results suggest that the overlap PA species, for example, 16:0/16:0-PA, has different targets specifically expressed in melanoma and T cells, respectively. Alternatively, nonoverlap PA species, for example, 16:1/16:2-PA, may selectively regulate T-cell functions.

In the present study, to explore how DGKα plays reverse roles in cancer cells and T lymphocytes, we searched for the target proteins of 16:0/16:0-PA in human melanoma cells. We identified heat shock protein (HSP) 27, which acts as a molecular chaperone and is a biomarker of cancer (36), as a novel SFA- and/or MUFA-containing PA (SFA/MUFA-PA)-binding protein. Moreover, 16:0/16:0-PA induced oligomer dissociation of HSP27, which is an indication of its activation. Furthermore, constitutively active DGKα recruited HSP27 to the plasma membrane and colocalized it in a DGK activity (PA)-dependent manner. Intriguingly, HSP27 protein was barely expressed in Jurkat T cells, while the protein was enriched in AKI melanoma cells. Therefore, these results strongly suggest that SFA- and/or MUFA-containing PA species generated by DGKα interact with HSP27 and selectively regulate its cancer-progressive function in melanoma cells but not in T cells.

## Results

### Identification of 16:0/16:0-PA-binding proteins in melanoma cells

**Figure 1:**
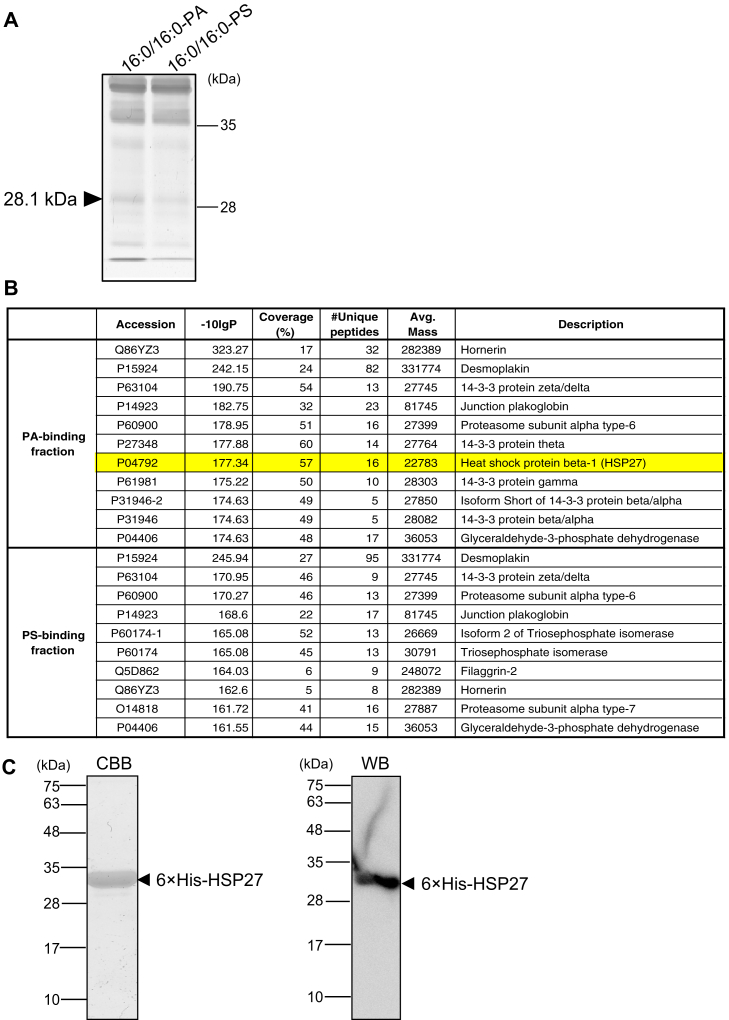
**Identification of HSP27 as a 16:0/16:0-PA target protein in melanoma cells**. *A*, AKI cell lysates were incubated with 16:0/16:0-PA-containing liposomes or 16:0/16:0-PS-containing liposomes for comparison and then ultracentrifuged. The 16:0/16:0-PA-binding and 16:0/16:0-PS-binding proteins were separated by SDS-PAGE and detected by silver staining. The band marked with a *black arrow* was excised, in-gel digested, and analyzed by LC-MS/MS. *B*, LC-MS/MS identified HSP27. The accession number of the protein registered in the UniProt FASTA database (*Accession*), probability of identification (–*10lgP*), the percentage of the protein sequence covered by identified peptides (*Coverage*), and the number of unique supporting peptides for the protein (*#Unique peptides*) are shown. *C*, the 6×His-HSP27 protein expressed in *E. coli* cells was purified, separated by SDS-PAGE (15% acrylamide), stained with Coomassie Brilliant Blue (*CBB*), and detected by Western blotting (*WB*) using an anti-6×His antibody. HSP, heat shock protein; LC-MS/MS, liquid chromatography-tandem mass spectrometry; PA, phosphatidic acid; PC, phosphatidylcholine; PS, phosphatidylserine; SDS-PAGE, sodium dodecyl sulfate polyacrylamide gel electrophoresis

We previously reported that DGKα preferentially generated SFA/MUFA-PA species, such as 16:0/16:0-, 16:0/18:0-, and 16:0/16:1-PA, in AKI human melanoma cells (30). In the present study, we chose 16:0/16:0-PA and sought its target proteins in AKI melanoma cells. 16:0/16:0-PA-containing liposomes and 16:0/16:0-phosphatidylserine (PS)-containing liposomes (as a control) were reacted with a soluble fraction of AKI cell lysates and then ultracentrifuged. An intense band with a molecular mass of approximately 28 kDa was found in the precipitates of 16:0/16:0-PA-liposomes by silver staining (Fig. 1*A*). By in-gel digestion and liquid chromatography/tandem mass spectrometry (LC-MS/MS) analysis, the proteins of this band were identified, and we focused on HSP27 (also known as HSPβ1, UniProt accession number: P04792, 205 aa), which was reproducibly detected in the precipitates of 16:0/16:0-PA-liposomes as a candidate 16:0/16:0-PA-binding protein from the ∼28 kDa band (Fig. 1*B*).

HSP27 is a molecular chaperone and is known to be a biomarker of cancer, renal injury and fibrosis, and neurodegenerative and cardiovascular disease (36). In particular, the levels of HSP27 are increased in hepatocellular carcinoma cells, and moreover, HSP27 promotes proliferation and invasion, which consequently confer aggressiveness to cancer cells (37, 38).

Next, we cloned human HSP27 cDNA (accession number: AB020027) from the mRNAs of AKI cells and ligated it with the pET-28a vector. 6× His-tagged HSP27 protein was produced in *Escherichia coli* cells and purified by affinity chromatography using nickel-nitrilotriacetic acid agarose. We confirmed that 6× His-HSP27 was detected as a single band with a molecular mass of approximately 30 kDa, which was recognized by an anti-6× His antibody and was thus successfully purified (Fig. 1*C*).

### HSP27 binds to 16:0/16:0-PA with high selectivity and affinity

**Figure 2:**
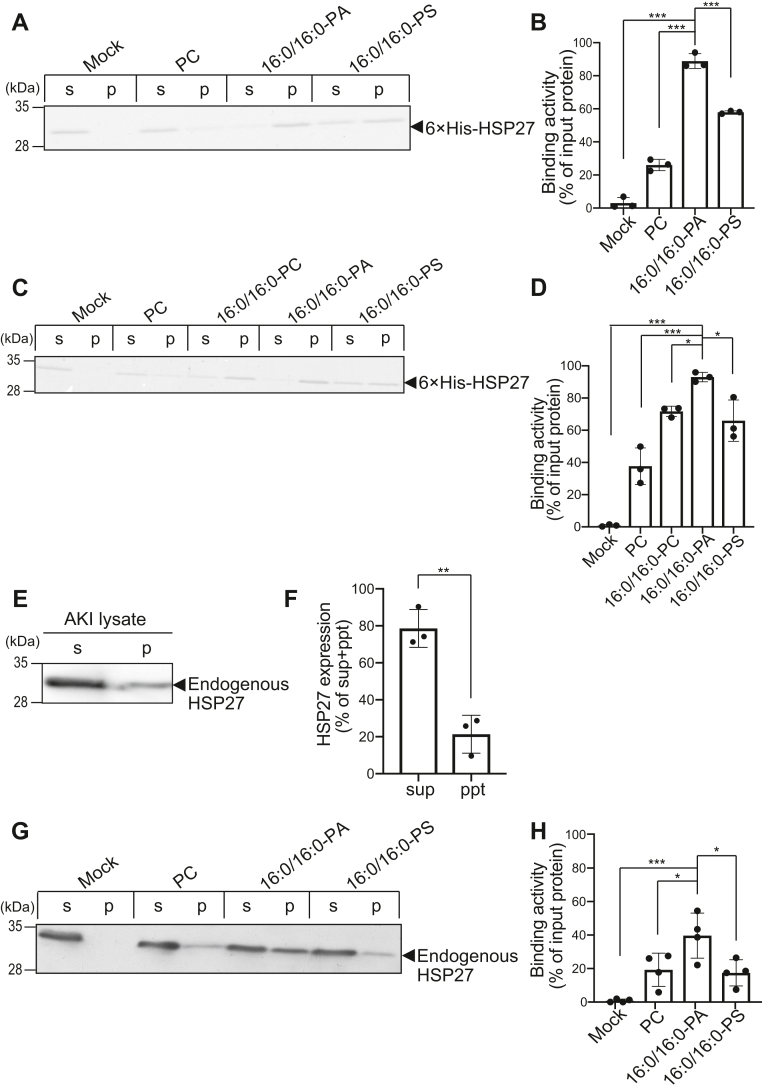
**Binding activity of 6×His-HSP27 to 16:0/16:0-PA.***A*, Liposome-binding assay of 6×His-HSP27 using 16:0/16:0-PA, 16:0/16:0-PS and PC liposomes was conducted. The purified 6×His-HSP27 (0.5 μM) was incubated with PC, 16:0/16:0-PA, or 16:0/16:0-PS liposomes (PA or PS: 200 μM) and then separated by ultracentrifugation. SDS-PAGE (15% acrylamide) was conducted, and separated proteins were stained with Coomassie Brilliant Blue. The position of 6×His-HSP27 is indicated with a *black arrowhead*. *C*, A liposome-binding assay of 6×His-HSP27 using 16:0/16:0-PA, 16:0/16:0-PS, 16:0/16:0-PC, and PC liposomes was conducted. The purified 6×His-HSP27 (1.0 μM) was incubated with PC, 16:0/16:0-PC, 16:0/16:0-PA, or 16:0/16:0-PS liposomes (PA or PS: 200 μM) and then separated by ultracentrifugation. SDS-PAGE (15% acrylamide) was conducted, and separated proteins were stained with Coomassie Brilliant Blue. The position of 6×His-HSP27 is indicated with a black arrowhead. *E*, AKI cells were washed two times with phosphate-buffered saline and lysed in HEPES buffer containing 25 mM HEPES (pH 7.4), 100 mM NaCl, and 1 mM dithiothreitol by sonication. After sonication, cell lysates were separated into soluble (supernatant (*s*)) and membrane (precipitate (*p*)) fractions by ultracentrifugation (200,000*g* for 30 min at 4 °C). The precipitate (*p*) was dissolved in HEPES buffer. SDS-PAGE (15% acrylamide) was conducted, and separated proteins were detected by Western blotting using an anti-HSP27 antibody. *F*, the amounts of protein in the supernatant (*s*) and precipitate (*p*) were quantified by densitometry using ImageJ software. HSP27 expression was calculated as the percentage of the supernatant or precipitate band intensity compared to the total band intensity. Values are presented as the mean ± SD of three independent experiments. ∗∗*p* < 0.01, two-tailed *t* test. *G*, Liposome-binding assay of endogenous HSP27 using 16:0/16:0-PA, 16:0/16:0-PS, and PC liposomes was conducted. AKI cell lysates were incubated with PC, 16:0/16:0-PA, or 16:0/16:0-PS liposomes (PA or PS: 200 μM) and then separated by ultracentrifugation. SDS-PAGE (15% acrylamide) was conducted, and separated proteins were detected by Western blotting using an anti-HSP27 antibody. The position of endogenous HSP27 is indicated with a black arrowhead. *B*, *D*, and *H*, The amounts of protein in the supernatant (*s*) and precipitate (*p*) were quantified by densitometry using ImageJ software. Binding activity was calculated as the percentage of the precipitate band intensity compared to the total band intensity. Values are presented as the mean ± SD of three independent experiments. ∗*p* < 0.05, ∗∗∗*p* < 0.005, one-way ANOVA followed by Tukey's post hoc test. ANOVA, analysis of variance; HSP, heat shock protein; PA, phosphatidic acid; PC, phosphatidylcholine; PS, phosphatidylserine; SDS-PAGE, sodium dodecyl sulfate polyacrylamide gel electrophoresis.

We next verified the interaction activity of 6 ×His-HSP27 with 16:0/16:0-PA using a liposome precipitation assay as described previously (39). Only approximately 25% of HSP27 was precipitated with liposomes containing phosphatidylcholine (PC) (neutral phospholipid) alone as a background control. Moreover, 16:0/16:0-PS-containing liposomes, as an acidic phospholipid control, cosedimented approximately 55% of HSP27 (Fig. 2, *A* and *B*). However, approximately 90% of HSP27 was sedimented with 16:0/16:0-PA-containing liposomes (Fig. 2, *A* and *B*), indicating that HSP27 more intensely binds to 16:0/16:0-PA liposomes than PC liposomes (background control) and 16:0/16:0-PS liposomes (acidic phospholipid control). Moreover, we analyzed 16:0/16:0-PC-binding activity of HSP27 and confirmed that it was almost the same with 16:0/16:0-PS (Fig. 2, *C* and *D*). Endogenous HSP27 expressed in AKI melanoma cells was mainly recovered in 200,000*g* sup (soluble fractions, ∼80%) (Fig. 2, *E* and *F*). The endogenous HSP27 recovered in soluble fractions also showed stronger interaction activity with 16:0/16:0-PA liposomes than 16:0/16:0-PS and PC liposomes (Fig. 2, *G* and *H*).

**Figure 3:**
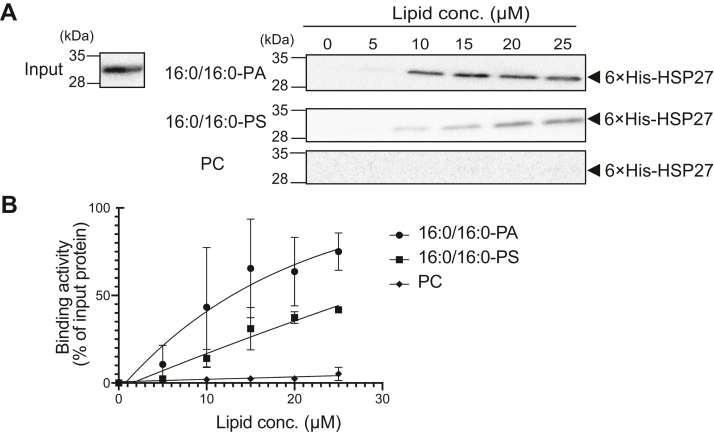
**Affinity of 6×His-HSP27 to 16:0/16:0-PA.***A*, Purified 6×His-HSP27 (0.1 μM) was incubated with the indicated concentrations (0–25 μM) of PC (control), 16:0/16:0-PS, and 16:0/16:0-PA liposomes and then separated by ultracentrifugation. SDS-PAGE (15% acrylamide) was conducted, and separated proteins were detected with Western blotting using an anti-6×His antibody. The position of 6×His-HSP27 is indicated with a *black arrowhead*. *B*, The amounts of protein in the precipitate were quantified by densitometry using ImageJ software. Binding activity was calculated as the percentage of the precipitate band intensity compared to the total band intensities (*input*). Values are presented as the mean ± SD of three independent experiments. The dissociation constant *K*_d_ was determined using GraphPad Prism 8 (a one-phase exponential decay model). HSP, heat shock protein; PA, phosphatidic acid; PC, phosphatidylcholine; PS, phosphatidylserine; SDS-PAGE, sodium dodecyl sulfate polyacrylamide gel electrophoresis.

We also determined the affinity of HSP27 for 16:0/16:0-PA by measuring binding activity at 0 to 25 μM PA. Liposome-binding HSP27 was augmented in a 16:0/16:0-PA and 16:0/16:0-PS concentration-dependent manner (Fig. 3*A*). However, HSP27 did not show marked binding activity to liposomes at 25 μM PC (Fig. 3*A*). The dissociation constant (*K*_d_) of HSP27 for 16:0/16:0-PA was determined to be 13.3 μM (Fig. 3*B*). The extrapolated *K*_d_ for 16:0/16:0-PS was calculated to be approximately 100 μM (Fig. 3*B*). These results indicate that HSP27 has a markedly higher affinity for PA than PS and PC.

**Figure 4:**
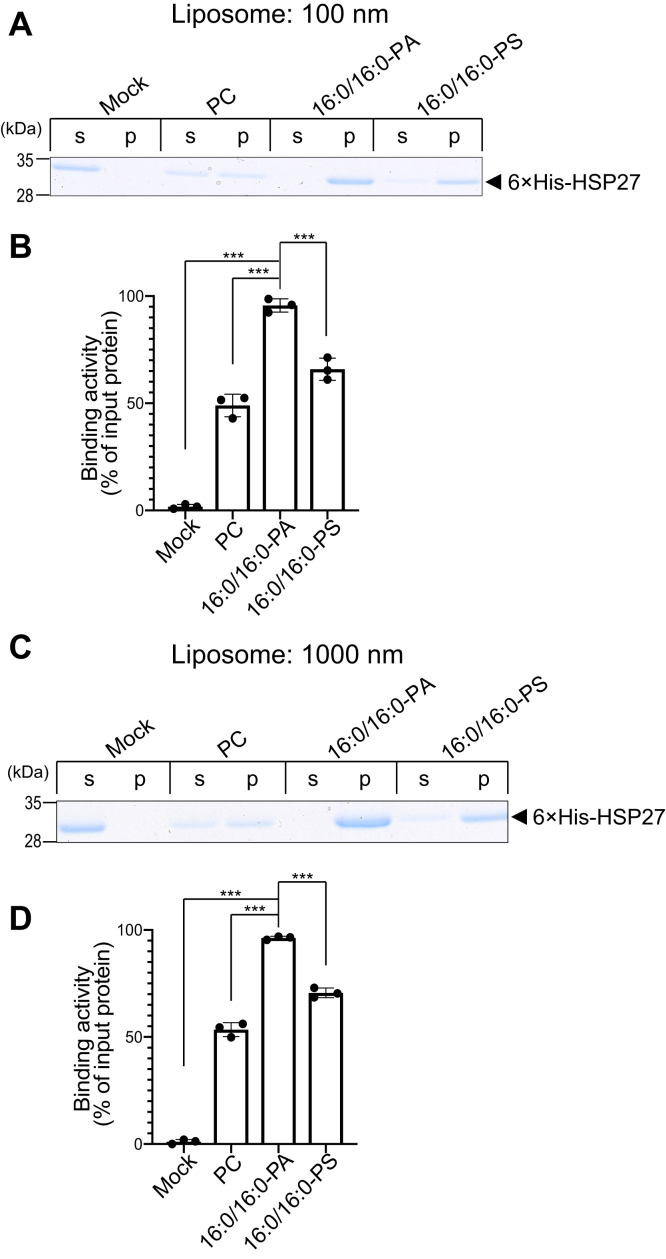
**Binding activity of 6×His-HSP27 to 16:0/16:0-PA in different liposome diameters.***A* and *C*, A liposome-binding assay of 6×His-HSP27 using 16:0/16:0-PA, 16:0/16:0-PS, and PC liposomes with different diameters (100 nm (*A*) and 1000 nm (*C*)) was conducted. The purified 6×His-HSP27 (0.5 μM) was incubated with PC, 16:0/16:0-PA or 16:0/16:0-PS liposomes (PA or PS: 200 μM) and then separated by ultracentrifugation. SDS-PAGE (15% acrylamide) was conducted, and separated proteins were stained with Coomassie Brilliant Blue. The position of 6×His-HSP27 is indicated with a black *arrowhead*. *B* and *D*, The amounts of protein in the supernatant (*s*) and precipitate (*p*) were quantified by densitometry using ImageJ software. Binding activity was calculated as the percentage of the precipitate band intensity compared to the total band intensity (100 nm (*B*) and 1000 nm (*D*)). Values are presented as the mean ± SD of three independent experiments. ∗∗∗*p* < 0.005, one-way ANOVA followed by Tukey's post hoc test. ANOVA, analysis of variance; HSP, heat shock protein; PA, phosphatidic acid; PC, phosphatidylcholine; PS, phosphatidylserine; SDS-PAGE, sodium dodecyl sulfate polyacrylamide gel electrophoresis.

The PA-binding activity of clathrin coat assembly protein AP180 was affected by liposome diameters (39). Therefore, the lipid binding activities of HSP27 were determined using liposomes with different diameters (100 nm and 1000 nm). HSP27 intensely bound to both sizes (100 nm (Fig. 4, *A* and *B*) and 1000 nm (Fig. 4, *C* and *D*)) of liposomes containing 16:0/16:0-PA), and the binding activity was stronger than those of 16:0/16:0-PS- and PC-containing liposomes (Fig. 4, *A*–*D*). No substantial differences between 100 nm and 1000 nm liposomes were observed (Fig. 4, *A*–*D*). Therefore, it is likely that different membrane curvatures and shapes formed by various liposome diameters fail to substantially affect the interaction of HSP27 with PA.

### HSP27 most strongly binds to PA among various lipids

**Figure 5:**
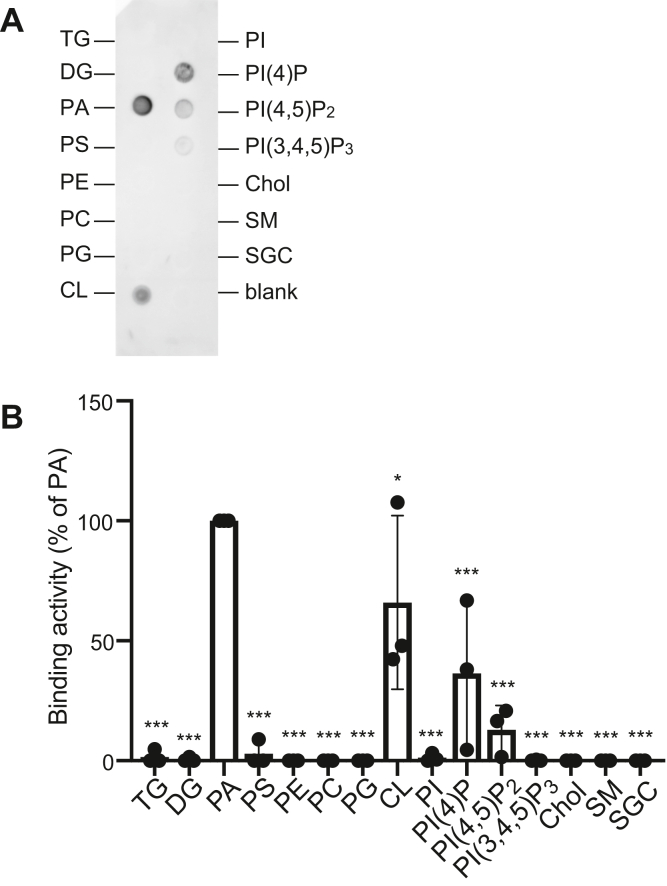
**Binding activity of 6×His-HSP27 to various lipids.***A*, Lipid overlay assay of 6×His-HSP27 using various lipids. Equimolar amounts (100 pmol) of various lipids were spotted onto nitrocellulose membranes (Lipid Strips, Echelon Biosciences) as indicated. The acyl chain(s) of these glycerolipids and sphingolipid are C16:0. The membrane was incubated with purified 6×His-HSP27 (20 nM). Lipid-bound proteins were detected with an anti-6×His antibody. The data shown are representative of three independent experiments that gave similar results. *B*, Spot intensities were quantified by densitometry using ImageJ software. The binding activity (spot intensity) of HSP27 to PA was set to 100%. Values are presented as the mean ± SD of three independent experiments. ∗*p* < 0.05, ∗∗∗*p* < 0.005 *versus* PA, one-way ANOVA followed by Tukey's post hoc test. ANOVA, analysis of variance; Chol, cholesterol; CL, cardiolipin; PA, phosphatidic acid; PC, phosphatidylcholine; PE, phosphatidylethanolamine; PG, phosphatidylglycerol; PI, phosphatidylinositol; PI(4)P, phosphatidylinositol-4-monophosphate; PI(4,5)P_2_, phosphatidylinositol-4,5-bisphosphate; PI(3,4,5)P_3_, phosphatidylinositol-3,4,5-trisphosphate; SGC, 3-sulfogalactosylceramide; SM, sphingomyelin; TG, triglyceride.

To measure the lipid binding selectivity of HSP27 in more detail, we carried out a lipid overlay assay using a nitrocellulose membrane spotted with diverse lipids that included 16:0 as fatty acid chains. HSP27 exhibited an intense interaction with 16:0/16:0-PA (Fig. 5*A*). However, other acidic lipids, such as phosphatidylinositol (PI), PS, phosphatidylglycerol, and 3-sulfogalactosylceramide, and neutral lipids, such as triglyceride, DG, PC, phosphatidylethanolamine, cholesterol (Chol), and sphingomyelin, failed to interact with HSP27 (Fig. 5*A*). PI-4-monophosphate (PI(4)P), PI-4,5-bisphosphate (PI(4,5)P_2_), and cardiolipin (CL), which are acidic lipids, are also associated with HSP27 (Fig. 5*A*). However, their binding intensities were lower than that of PA (Fig. 5*B*). Therefore, these results indicate that HSP27 selectively and most intensely binds to PA.

### HSP27 strongly interacts with SFA/MUFA-PA but not PUFA-PA

**Figure 6:**
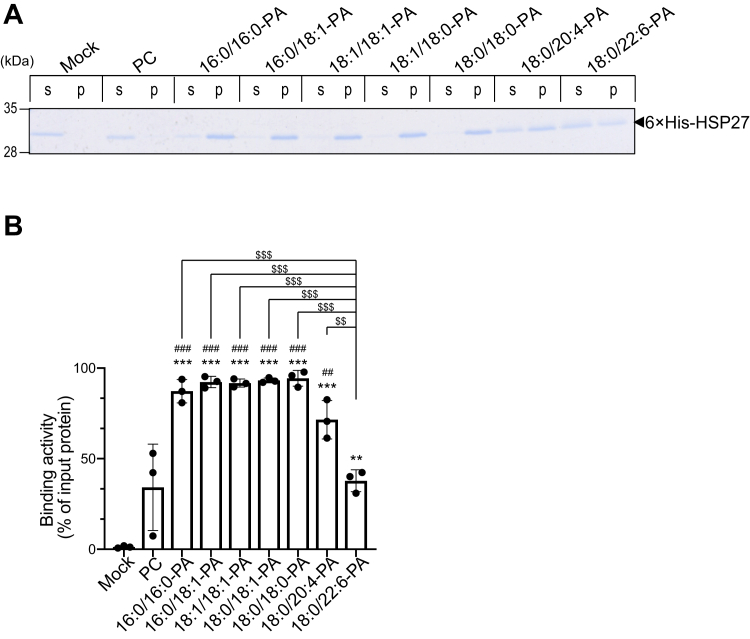
**Binding activity of 6×His-HSP27 to various PA species**. *A*, purified 6×His-HSP27 (0.5 μM) was incubated with PC, 16:0/16:0-PA, 16:0/18:1-PA, 18:1/18:1-PA, 18:0/18:1-PA, 18:0/18:0-PA, 18:0/20:4-PA or 18:0/22:6-PA liposomes (PA: 200 μM) and then separated by ultracentrifugation. SDS-PAGE (15% acrylamide) was conducted, and separated proteins were stained with Coomassie Brilliant Blue. The position of 6×His-HSP27 is indicated with a black arrowhead. *B*, the amounts of protein in the supernatant (*s*) and precipitate (*p*) were quantified by densitometry using ImageJ software. Binding activity was calculated as the percentage of the precipitate band intensity compared to the total band intensity. Values are presented as the mean ± SD of three independent experiments. ∗∗*p* < 0.01, ∗∗∗*p* < 0.005, one-way ANOVA followed by Tukey's post hoc test. ANOVA, analysis of variance; HSP, heat shock protein; PA, phosphatidic acid; PC, phosphatidylcholine.

To assess the PA molecular species selectivity of HSP27, we performed a liposome precipitation assay using various PA species, including 16:0/16:0-, 16:0/18:1-, 18:1/18:1-, 18:0/18:1-, 18:0/18:0-, 18:0/20:4- and 18:0/22:6-PA. SFA- and/or MUFA-containing PAs, 16:0/18:1-, 18:1/18:1-, 18:0/18:1- and 18:0/18:0-PA, intensely interacted with HSP27 (Fig. 6*A*). The binding activities of these PA species were almost the same as that of 16:0/16:0-PA (Fig. 6*B*). However, PUFA-containing PAs, 18:0/20:4- and 18:0/22:6-PA, showed substantially lower binding activities to HSP27 (Fig. 6*B*). These results indicate that HSP27 preferably binds to SFA- and/or MUFA-containing PAs.

### 16:0/16:0-PA-binding activities of N- and C-terminal regions of HSP27

**Figure 7:**
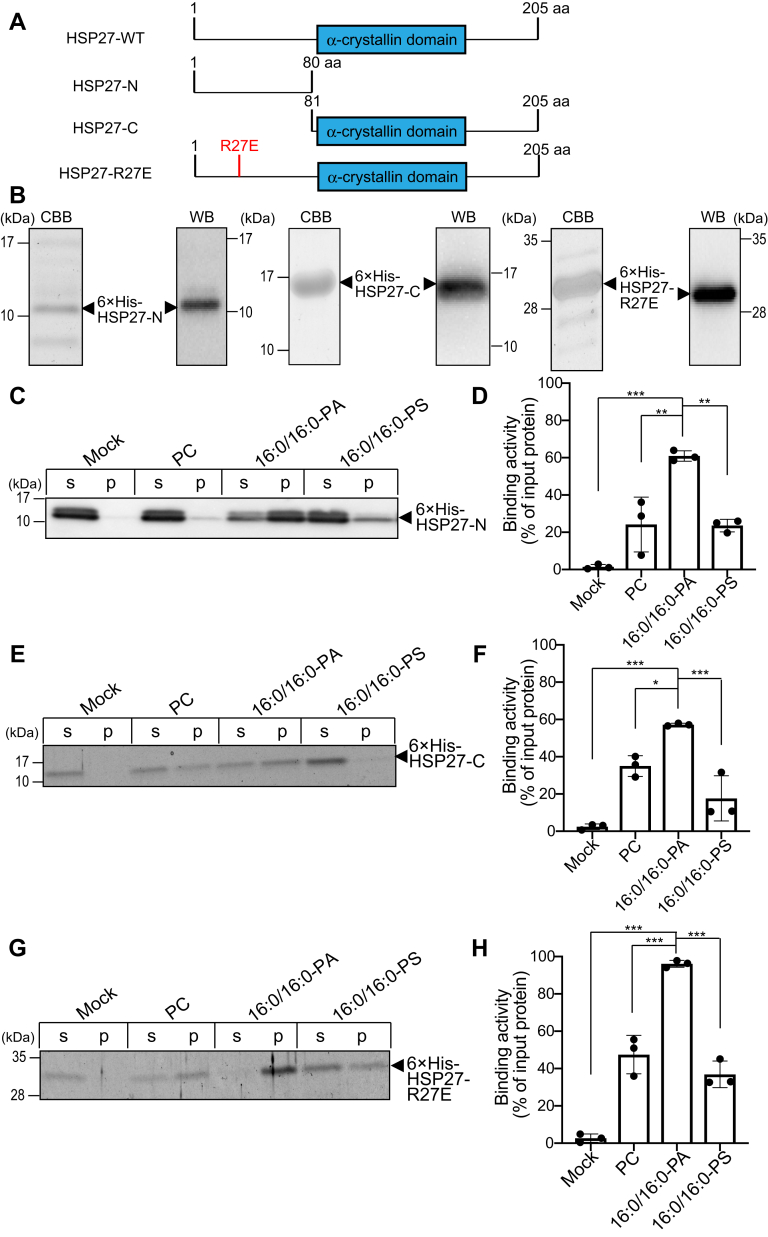
**Binding activities of 6×His-HSP27 and its mutants to 16:0/16:0-PA.***A* and *B*, The 6×His-HSP27-N (1–80 aa), 6×His-HSP27-C (81–205 aa), and 6×His-HSP27-R27E proteins expressed in *E. coli* cells (*A*) were purified, separated by SDS-PAGE (15% acrylamide), stained with Coomassie *Brilliant Blue*, and detected by Western blotting with anti-6×His antibody (*B*). *C*, *E*, and *G*, Liposome binding assay of 6×His-HSP27-N (*C*), 6×His-HSP27-C (*E*), and 6×His-HSP27-R27E using 16:0/16:0-PA, 16:0/16:0-PS and PC liposomes (X/PC/chol = 20/50/30 mol% (X = PC, PA or PS) (1 mM total lipid). The purified (*C*) 6×His-HSP27-N (1.1 μM), (*E*) 6×His-HSP27-C (1.1 μM) or (*G*) 6×His-HSP27-R27E (0.5 μM) was incubated with each liposome (1 mM total lipids) and then separated by ultracentrifugation. SDS-PAGE (15% acrylamide) was performed, and separated proteins were detected by Western blotting with anti-6×His antibody (*C*) or stained with Coomassie *Brilliant Blue* (*E* and *G*). *D*, *F*, and *H*, the amounts of protein in the supernatant (*s*) and precipitate (*p*) were quantified by densitometry using ImageJ software. Binding activity was calculated as the percentage of the precipitate band intensity compared to the total band intensity. Values are presented as the mean ± SD of three independent experiments. ∗*p* < 0.05, ∗∗*p* < 0.01, ∗∗∗*p* < 0.005, one-way ANOVA followed by Tukey's post hoc test. ANOVA, analysis of variance; HSP, heat shock protein; PA, phosphatidic acid; PC, phosphatidylcholine; PS, phosphatidylserine; SDS-PAGE, sodium dodecyl sulfate polyacrylamide gel electrophoresis.

To determine the 16:0/16:0-PA-binding region in HSP27, we divided HSP27 into two parts, the N-terminal region (HSP27-NT, aa 1–80) and the C-terminal region (HSP27-CT, aa 81–205) of HSP27 including an α-crystallin domain (Fig. 7*A*), and expressed in *E. coli* and highly purified them (Fig. 7*B*). HSP27-NT interacted moderately more strongly with 16:0/16:0-PA than HSP27-CT (Fig. 7, *C* and *D*), indicating that while the N-terminal region more strongly contributes to the 16:0/16:0-PA binding, both the N- and C-terminal regions of HSP27 are important for 16:0/16:0-PA binding.

We next attempted to determine residues in HSP27 critical for 16:0/16:0-PA binding. Some reports have indicated that α-synuclein has α-helices containing several basic residues, which bind to acidic phospholipids (40, 41, 42). We searched predicted α-helices in HSP27 using Chou and Fasman secondary structure prediction (https://www.biogem.org/tool/chou-fasman/) and found a sole Arg-containing α-helix (aa 26–32) in HSP27-NT (aa 1–80). Therefore, Arg27 was replaced with Glu (HSP27-R27E) (Fig. 7*A*), expressed in *E. coli*, and highly purified (Fig. 7*B*). However, the 16:0/16:0-PA-binding activity of HSP27-R27E was not considerably reduced (Fig. 7, *G* and *H*). These results indicate that Arg27 is not a critical residue for the 16:0/16:0-PA binding of HSP27.

### HSP27 colocalizes with constitutively active DGKα in cells

**Figure 8:**
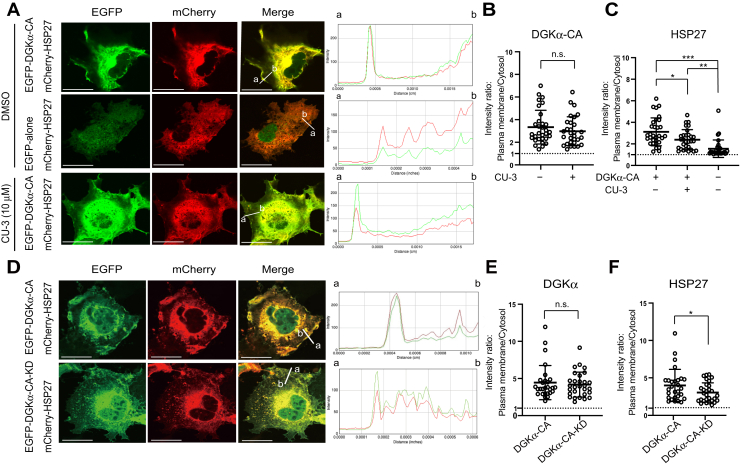
**Translocation of HSP27 from the cytoplasm to the plasma membrane depending on DGKα-DGKα-CA**. *A*, EGFP alone or EGFP-DGKα-CA (a constitutively active mutant) was co-expressed with mCherry-HSP27 in COS-7 cells as indicated. After 20 h of transfection, 10 μM CU-3 (a DGKα-selective inhibitor) or DMSO was added and incubated for 30 min, and then the cells were fixed. The localization of EGFP-DGKα-CA (*green*) and mCherry-HSP27 (*red*) in the absence or presence of CU-3 was quantified using ImageJ software. Bars, 25 μm. *B*, quantitative image analysis of EGFP-DGKα-CA localization at the plasma membrane in COS-7 cells in the presence of DMSO (n = 31 from three independent experiments, 10–11 cells/experiment) or CU-3 (n = 30 from three independent experiments, 10 cells/experiment). Each dot shows the plasma membrane: cytosol intensity ratio for EGFP-DGKα-CA in COS-7 cells. Values are presented as the mean ± SD. Two-tailed *t* test. *C*, quantitative image analysis of mCherry-HSP27 localization cotransfected with pEGFP DGKα-CA or pEGFP alone at the plasma membrane in COS-7 cells in the presence of DMSO (n = 31 from three independent experiments, 10–11 cells/experiment (cotransfected with pEGFP-DGKα-CA or pEGFP-alone)) or CU-3 (n = 30 from three independent experiments, 10 cells/experiment). Each dot shows the plasma membrane: cytosol intensity ratio mCherry-HSP27 in COS-7 cells. Values are presented as the mean ± SD. ∗*p* < 0.05, ∗∗*p* < 0.01, ∗∗∗*p* < 0.005, one-way ANOVA followed by Tukey’s post hoc test. *D*, EGFP-DGKα-CA or EGFP-DGKα-CA-KD (a kinase-dead mutant) was co-expressed with mCherry-HSP27 in COS-7 cells as indicated. After 20 h of transfection, the cells were fixed. The localization of EGFP-DGKα-CA (*green*), EGFP-DGKα-CA-KD (*green*), and mCherry-HSP27 (*red*) was quantified using ImageJ software. Bars, 25 μm. *E*, Quantitative image analysis of EGFP-DGKα-CA (n = 28 from three independent experiments, 9–10 cells/experiment) and EGFP-DGKα-CA-KD (n = 29 from three independent experiments, 9–10 cells/experiment) localization at the plasma membrane in COS-7 cells. Each dot shows the plasma membrane:cytosol intensity ratio for EGFP-DGKα-CA and EGFP-DGKα-CA-KD in COS-7 cells. Values are presented as the mean ± SD. Two-tailed *t* test. *F*, quantitative image analysis of mCherry-HSP27 (cotransfected with pEGFP DGKα-CA (n = 28 from three independent experiments, 9–10 cells/experiment) or pEGFP-DGKα-CA-KD (n = 29 from three independent experiments, 9–10 cells/experiment) localization at the plasma membrane in COS-7 cells. Each dot shows the plasma membrane: cytosol intensity ratio mCherry-HSP27 in COS-7 cells. Values are presented as the mean ± SD. ∗*p* < 0.05, two-tailed *t* test. ANOVA, analysis of variance; DGK, diacylglycerol kinase; HSP, heat shock protein; PA, phosphatidic acid; PC, phosphatidylcholine; PS, phosphatidylserine; SDS-PAGE, sodium dodecyl sulfate polyacrylamide gel electrophoresis.

We next investigated whether HSP27 can associate with PA in cells. When EGFP-DGKα-CA (a constitutively active mutant lacking EF-hand motifs (Δ1–196)), which produces SFA- and/or MUFA-containing PAs (16:0/18:0-, 16:0/18:1- and 18:0/18:1-PA) (29), was expressed in COS-7 cells, the constitutively active mutant was located at the plasma membrane (Fig. 8, *A*–*C*). Moreover, mCherry-HSP27 coexpressed with EGFP-DGKα-CA showed a higher plasma membrane/cytosol ratio of HSP27 at the membrane region where DGKα-CA is colocalized with HSP27 (∼3.1) than mCherry-HSP27 coexpressed with EGFP alone (∼1.6) (Fig. 8, *A*–*C*). Notably, the plasma membrane/cytosol ratio (∼2.4) of HSP27 was significantly attenuated by CU-3 (10 μM), a DGKα-selective inhibitor (29), (Fig. 8, *A* and *C*), although DGKα-CA was located at the plasma membrane even in the presence of CU-3 (Fig. 8, *A* and *B*). These results indicate that the colocalization of EGFP-DGKα-CA with HSP27 was markedly reduced by CU-3. In addition, although EGFP-DGKα-CA-KD, a kinase-dead inactive mutant of DGKα-Δ1–196 in which Gly-435 is substituted with Asp (43), was located at the plasma membrane (plasma membrane/cytosol ratio: ∼4.0) (Fig. 8, *D* and *E*), plasma membrane/cytosol ratio (∼3.0) of mCherry-HSP27 coexpressed with the inactive mutant was lower than that (∼4.0) with EGFP-DGKα-CA (Fig. 8, *D* and *F*). These results indicate that the inactive mutant was less strongly colocalized with mCherry-HSP27 than EGFP-DGKα-CA. Therefore, these results indicate that the colocalization between HSP27 and DGKα occurs in a DGK activity (PA production)-dependent manner, suggesting that HSP27 can interact with PA produced by DGKα in cells.

### 16:0/16:0-PA induces dissociation of the HSP27 oligomer

**Figure 9:**
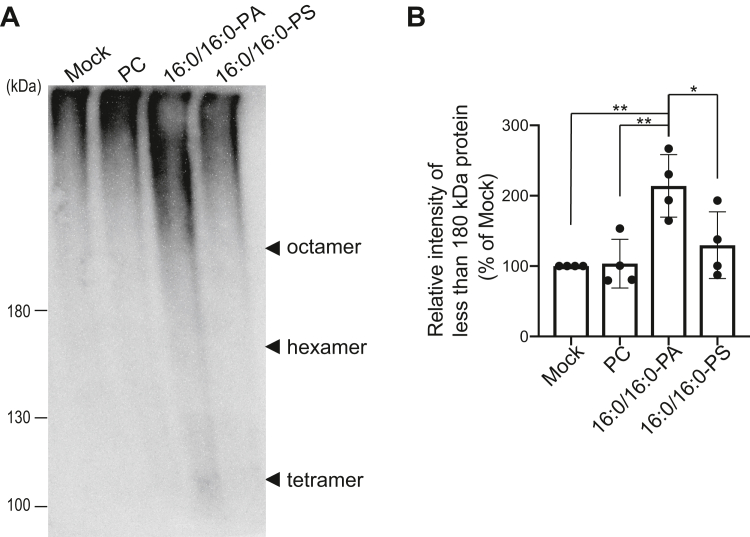
**Oligomer dissociation of HSP27 depending on 16:0/16:0-PA**. *A*, purified 6×His-HSP27 (2.3 μM) was incubated with 16:0/16:0-PA, 16:0/16:0-PS, and PC liposomes (PA or PS: 200 μM). BN-PAGE (4% stacking gel, 6% separation gel) was conducted, and separated proteins were detected with Western blotting using an anti-6×His antibody. The positions of tetramer, hexamer, and octamer of 6×His-HSP27 are indicated with black arrowheads. *B*, the amounts of less than 180 kDa protein were quantified by densitometry using ImageJ software. The band intensity of less than 180 kDa protein of mock was set to 100%. Values are presented as the mean ± SD of four independent experiments. ∗*p* < 0.05, ∗∗*p* < 0.01, one-way ANOVA followed by Tukey’s post hoc test. ANOVA, analysis of variance; BN-PAGE, blue native sulfate polyacrylamide gel electrophoresis; HSP, heat shock protein; PA, phosphatidic acid; PC, phosphatidylcholine; PS, phosphatidylserine.

It was reported that HSP27 forms a large oligomer (12-mer–35-mer) (44) and that the oligomer dissociation of HSP27 enhances its chaperone activity (44, 45). To assess oligomer dissociation, we performed blue native polyacrylamide gel electrophoresis (BN-PAGE). HSP27 was detected as a large oligomer (more than octamer) in the absence of phospholipids (Fig. 9*A*). In the presence of PC and 16:0/16:0-PS, HSP27 was electrophoresed to the same position as that in the absence of phospholipids (Fig. 9). However, in the presence of 16:0/16:0-PA, HSP27 was partly detected as less than octamer (tetramer and hexamer) (Fig. 9), suggesting that 16:0/16:0-PA selectively induces oligomer dissociation of HSP27.

### HSP27 is highly expressed in AKI melanoma cells but not Jurkat T cells

**Figure 10:**
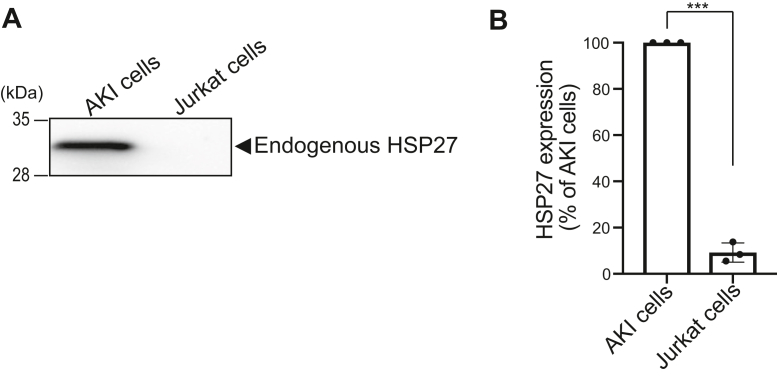
**HSP27 expression in AKI and Jurkat cells.***A*, AKI cells and Jurkat cells were washed two times with phosphate-buffered saline and lysed in HEPES buffer containing 25 mM HEPES (pH 7.4), 100 mM NaCl, and 1 mM dithiothreitol by sonication. After sonication, cell lysates were separated into soluble (supernatant (*s*)) and membrane (precipitate (*p*)) fractions by ultracentrifugation (200,000*g* for 30 min at 4 °C). The precipitate (*p*) was dissolved in HEPES buffer. SDS-PAGE (15% acrylamide) was conducted and separated proteins were detected by Western blotting using an anti-HSP27 antibody. *B*, the amounts of protein in AKI cells and Jurkat cells were quantified by densitometry using ImageJ software. HSP27 expression was calculated as the percentage of the band intensity of AKI lysates or Jurkat lysates compared to the band intensity of AKI lysates. Values are presented as the mean ± SD of three independent experiments. ∗∗∗*p* < .005, two-tailed *t* test. ANOVA, analysis of variance; HSP, heat shock protein; SDS-PAGE, sodium dodecyl sulfate polyacrylamide gel electrophoresis.

To analyze whether HSP27 is substantially expressed in T cells, where DGKα plays roles different from cancer cells (6, 34), we next examined the expression levels of HSP27 in Jurkat T cells by Western blotting. We found that the HSP27 protein was barely detectable in Jurkat T cells (less than 10% of AKI melanoma cells) (Fig. 10, *A* and *B*), although the protein band was strongly detected in AKI melanoma cells (Fig. 10, *A* and *B*), suggesting that SFA/MUFA-PAs function *via* HSP27 only in melanoma cells but not in T cells.

## Discussion

In the present study, we demonstrated for the first time that a chaperone, HSP27, is a novel PABP that prefers SFA- and/or MUFA-containing PA species. Several PABPs have been reported to recognize different PA species (11, 12, 13, 14, 15). HSP27 was newly added to the list, which is still growing. Active DGKα recruited and colocalized with HSP27 at the plasma membrane in a DGK activity (PA)-dependent manner. Notably, 16:0/16:0-PA induced oligomer dissociation of HSP27, which enhances its chaperone activity.

HSP27 most strongly interacted with PA among the lipids examined (Fig. 5). However, HSP27 interacted with CL, PI(4)P, and PI(4,5)P_2_ with lower affinity than PA (Fig. 5). HSP27, which is a cytosolic protein, can interact with PA, PI(4)P, and PI(4,5)P_2_ but not with CL because CL is primarily localized in the inner membranes of mitochondria (46). The amounts of PI(4)P and PI(4,5)P_2_ are substantially low in cells (0.02–0.2 mol% of total cellular phospholipids) (47) compared with PA (1–4 mol% of total cellular phospholipids) (48). Therefore, it is likely that HSP27 acts as a PABP, but not a CL- or phosphoinositide-binding protein, in cells.

Essentially the same results (PA, PC, and PS binding) were obtained by the liposome sedimentation (Figs. 2 and 4) and lipid overlay (Fig. 5) assays. However, high background activities, probably due to hydrophobic interaction, were observed in the liposome sedimentation assay. It is likely that, in the lipid overlay assay, HSP27 mainly recognizes the hydrophilic polar head of PA. However, in the liposome sedimentation assay, HSP27 probably distinguishes fatty acid moieties (hydrophobic region) of PA in liposomes and/or different circumstances on liposome surfaces including different density of lipids generated by distinct fatty acid composition of PAs in addition to the polar head of PA.

The amounts of PS are higher than those of PA in cellular membranes. PS liposomes cosedimented HSP27 (Fig. 2, *G* and *H*). However, the sedimentation activity is almost the same as PC-binding activity (Fig. 2, *G* and *H*). Moreover, the PS-binding activity was not detected in the lipid overlay assay (Fig. 5). Indeed, HSP27 is not localized to the plasma membrane where PS is enriched (Fig. 8). Moreover, PS did not induce oligomer dissociation of HSP27 (Fig. 9). These results allow us to speculate that PS-binding activity of HSP27 is substantially lower than its PA-binding activity and does not activate HSP27 in cells.

The *K*_d_ of HSP27 for 16:0/16:0-PA was determined to be 13.3 μM (Fig. 3). The *K*_d_ values of PDE4A1, Opi1p, and sporulation-specific protein 20p for PA are 6.8, 4.5, and 2.2 μM, respectively (49). In addition, the values of α-synuclein (50), creatine kinase muscle type (51), L-lactate dehydrogenase A (52), synaptojanin-1 (53) and neurofibromatosis type-1 (Ras GTPase-activating protein) (54) were 6.6, 2.0, 3.8, 0.5, and 12.0 μM, respectively. Therefore, the value of HSP27 is comparable to those of the PABPs described earlier.

HSP27 binds to SFA/MUFA-PA more strongly than to PUFA-PA (Fig. 6). There is only one report indicating that a PABP, creatine kinase muscle type, prefers SFA/MUFA-PAs (51). In contrast to these PABPs, PDE4A1, Opi1p, and sporulation-specific protein 20p failed to exhibit substantial preference among PA molecular species (49). α-Synuclein prefers MUFA-containing PA (18:1/18:1-PA) over SFA-containing PAs (16:0/16:0- and 18:0/18:0-PA) and PUFA-containing PAs (18:0/20:4-PA) (55). L-lactate dehydrogenase A interacted with SFA-containing and PUFA-containing PAs more strongly than MUFA-containing PAs (52). Praja-1 (56), synaptojanin-1 (53), and clathrin coat assembly protein AP180 (39) preferentially bound to PUFA-PA. DGKγ more intensely associates with 18:0/20:4-PA and 18:1/18:1-PA than 18:0/18:0-PA (57). These results indicate that different PABPs display selectivities for different molecular species of PA.

It is possible that, in addition to DGKα, other PA-producing enzymes such as lysoPA acyltransferase and phospholipase D (PLD) also affect HSP27 function. Indeed, lysoPA acyltransferase isoform expression has been shown to enhance the proliferation of cancer cells and correlates with an increased risk of tumor development and aggressiveness of tumors (58). Moreover, increased expression of PLD enzymes (PLD1 and PLD2) has been implicated as contributing factors in several types of human cancer, and the role of PLD in pathways involved in cancer progression and tumorigenesis has been reported (59).

Although HSP27 exists as a large oligomer (12-mer–35-mer) (44), Ser/Thr phosphorylation destabilizes HSP27 oligomeric assembly and leads to a dimer form, which is active (44, 45). Moreover, oligomer decomposition of purified HSP27 by reduction, even without phosphorylation, also results in activation (60), indicating that deoligomerization itself induces HSP27 activation. Notably, 16:0/16:0-PA, but not PC or 16:0/16:0-PS, also selectively induced oligomer dissociation of HSP27 (Fig. 9). Therefore, it is likely that SFA-/MUFA-PA-dependent deoligomerization causes activation of HSP27.

PA binding may have effects similar to Ser/Thr phosphorylation because both PA binding and phosphorylation introduce negative charge(s) of a phosphate group to the protein. It was reported that HSP27 is phosphorylated by mitogen-activated protein kinase (MAPK)-activated protein kinase (MAPKAPK) 2 and 3, MAPKAPK5, PKC, cGMP-dependent kinase, Akt/protein kinase B, and protein kinase D (61). The activities of PKCδ (62) and ε (63, 64) are enhanced by PA in addition to DG. Akt/protein kinase B (65, 66) and C-Raf upstream of MAPK (extracellular signal-regulated kinase (ERK)) (67, 68, 69) are also activated by PA. Therefore, it is possible that PA synergistically induces the dissociation of HSP27 oligomers *via* direct binding to HSP27 and activation of HSP27 phosphorylation pathways.

Overexpressed DGKα recruited HSP27 to the plasma membrane in COS7 cells in a DGK activity (PA)-dependent manner (Fig. 8), suggesting that SFA- and/or MUFA-containing PA species generated by DGKα bind to and recruit HSP27 to the plasma membrane and activate the protein. DGKα is highly expressed in cancer cells, such as melanoma (23) and hepatocellular carcinoma (24) cells, but not in normal melanocytes or hepatocytes. Therefore, it is possible that SFA- and/or MUFA-containing PAs produced by abundant DGKα can interact with and activate HSP27 in cancer cells as well and, consequently, induce cancer cell proliferation and cancer progression. DGKα commonly generates SFA- and/or MUFA-containing PA species, for example, 16:0/16:0-PA in melanoma and T cells (30, 35). However, HSP27 is barely expressed in Jurkat T cells (Fig. 10), while HSP27 is also enriched in cancer cells (37, 38). Moreover, the human protein atlas showed that HSP27 is not expressed in lymph node and spleen (https://www.proteinatlas.org/ENSG00000106211-HSPB1/tissue). Therefore, this expression pattern may explain at least in part how DGKα plays reverse roles in T cells (attenuator) and cancer cells (enhancer) (6, 34). However, further studies are required to elucidate signal transduction through the DGKα–16:0/16:0-PA–HSP27 pathway during cancer cell proliferation and cancer progression more in detail and how DGKα has cancer- and T-cell-selective functions.

Recently, it has been revealed that DGK isozymes utilize different DG species and thus produce distinct PA species in different cells (12, 70). DGKα generates SFA- and/or MUFA-containing PAs, such as 16:0/16:0-, 16:0/18:0-, and 16:0/16:1-PA, in serum-starved AKI melanoma cells (30). In the present study, we demonstrated that SFA/MUFA-PAs selectively interacted with HSP27 (Fig. 6) and enhanced its oligomer dissociation (Fig. 7), which is closely linked to its activation (44, 45). Indeed, colocalization of DGKα and HSP27 at the plasma membrane was observed (Fig. 8). Moreover, a DGKα-selective inhibitor attenuated the colocalization (Fig. 8), indicating that the colocalization occurred in a DGK activity (PA)-dependent manner. Therefore, there is the possibility that SFA/MUFA-PAs produced by DGKα activate HSP27 in cancer cells.

Elevated levels of DGKα and PA are related to cancer initiation and progression (23, 34, 71, 72). DGKα prevents apoptosis through the PKCζ–NF-κB pathway in melanoma cells (23). Moreover, DGKα promotes hepatocellular carcinoma proliferation *via* activation of the Ras–Raf–MAPK/ERK kinase–ERK pathway (24). Furthermore, this isozyme inhibits apoptosis of glioblastoma and melanoma cells through the PDE4A1–cAMP–mTOR pathway (32). PKCζ (73), C-Raf (67, 68, 69), and PDE4A1 (49, 74) are activated by PA. Therefore, it is likely that PA regulates the activities of these enzymes in the signaling pathways. In addition to these enzymes, our results showed that PA targets HSP27, which prevents apoptosis and promotes proliferation in cancer cells. Therefore, it is possible that DGKα utilizes multiple pathways to promote the aggressiveness of cancer cells.

In summary, in the present study, we demonstrated that SFA/MUFA-PAs, which are produced by DGKα in cancer cells (30), strongly bind to HSP27, which is highly expressed in melanoma cells but not in T cells and attenuates its oligomer formation. Our results shed light on a novel function of SFA/MUFA-PA and allow us to speculate about the functional linkage between the pro-cancer proteins, HSP27 (37, 38) and DGKα (33, 34).

## Experimental procedures

### Materials

L-α-PC from egg yolk, 1,2-dipalmitoyl-sn-glycero-3-phosphoserine (16:0/16:0-PS), 1,2-dipalmitoyl-sn-glycero-3-phosphate (16:0/16:0-PA), 1,2-distearoyl-sn-glycero-3-phosphate (18:0/18:0-PA), 1-palmitoyl-2-oleoyl-sn-glycero-3-phosphate (16:0/18:1-PA), 1-stealoyl-2-oleoyl-sn-glycero-3-phosphate (18:0/18:1-PA), 1,2-dioleoyl-sn-glycero-3-phosphate (18:1/18:1-PA), 1-stearoyl-2-arachidonoyl-sn-glycero-3-phosphate (18:0/20:4-PA), and 1-stearoyl-2-docosahexaenoyl-sn-glycero-3-phosphate (18:0/22:6-PA) were purchased from Avanti Polar Lipids (Alabaster, AL, USA). Cholesterol (Chol) was purchased from Wako Pure Chemical Industries (Tokyo, Japan). Membrane Lipid Strips were purchased from Echelon Biosciences (Salt Lake City, UT, USA).

### Cell culture

AKI cells (a human melanoma-derived cell line) and COS-7 cells (fibroblast-like cell lines derived from monkey kidney tissue) were grown in Dulbecco’s modified Eagle’s medium (DMEM; Wako Pure Chemical Industries) supplemented with 10% fetal bovine serum (Thermo Fisher Scientific, Waltham, MA), 100 units/ml penicillin, and 100 μg/ml streptomycin (Wako Pure Chemical Industries). The cells were maintained at 37 °C in an environment containing 5% CO_2_.

### Preparation of liposomes

The following lipid mixtures were used to identify 16:0/16:0-PA-specific binding proteins from AKI melanoma cells: 16:0/16:0-PS liposomes [Chol (30 mol%) and PC Mix (from egg yolk) (60 mol%) and 16:0/16:0-PS (10 mol%)] and 16:0/16:0-PA liposomes [Chol (30 mol%) and PC Mix (from egg yolk) (60 mol%) and 16:0/16:0-PA (10 mol%)]. The combined dried lipid mixture was hydrated at 95 °C in HEPES buffer (25 mM HEPES, pH 7.4, 100 mM NaCl, and 1 mM dithiothreitol) for 45 min and vortexed for 1 min once every 15 min during hydration. The liposomes were then subjected to five freeze–thaw cycles (−196 °C for 3 min, 95 °C for 3 min) (75). Liposomes were formed by sonication at 90 °C using a Branson Sonifier 450 (76).

The following lipid mixtures were used to determine the properties of the HSP27 protein: the control liposome [Chol (30 mol%) and PC Mix (from egg yolk) (70 mol%)], PS liposome [Chol (30 mol%), PC Mix (from egg yolk) (50 mol%) and 16:0/16:0-PS (20 mol%)], and the PA liposome [Chol (30 mol%), PC Mix (from egg yolk) (50 mol%) and each PA species (20 mol%)]. For the lipid-binding assay, the combined dried lipid mixture was hydrated at 95 °C in HEPES buffer for 45 min and vortexed for 1 min once every 15 min during hydration. The liposomes were then subjected to five freeze–thaw cycles (−196 °C for 3 min, 95 °C for 3 min). Liposomes were formed by sonication at 90 °C using a Branson Sonifier 450, or the liposomes were further extruded 11 times through a 100 nm or 1000 nm polycarbonate membrane using a Mini Extruder (Avanti Polar Lipids) (77). The extruder was brought to 95 °C prior to extrusion. Since the lipid forms a bilayer, half of the actual concentration was considered (78).

### Identification of HSP27 as a PA-binding protein

AKI cells were washed two times with phosphate-buffered saline and lysed in HEPES buffer containing 25 mM HEPES (pH 7.4), 100 mM NaCl, and 1 mM dithiothreitol. After sonication, insoluble materials were removed by ultracentrifugation (200,000*g* for 30 min at 4 °C). AKI cell lysates were incubated with the PC liposomes at 4 °C for 30 min, and nonspecific protein bound to the vesicles were removed by centrifugation at 200,000*g* for 1 h at 4 °C. The resultant supernatant was incubated with the 16:0/16:0-PS or 16:0/16:0-PA liposomes at 4 °C for 30 min and then centrifuged at 200,000*g* at 4 °C for 1 h. The precipitates were dissolved in HEPES buffer containing 25 mM HEPES, pH 7.4, 100 mM NaCl, and 1 mM dithiothreitol. The 16:0/16:0-PA-binding proteins were separated by SDS-PAGE and visualized by silver staining (Fig. 1). In-gel digestion and LC-MS/MS identification of proteins in the ∼28 kDa bands were carried out as previously described (79). Desalted tryptic peptides were analyzed by an Ultimate 3000 RSLCnano system (Thermo Fisher Scientific, Waltham, MA, USA) coupled to a Q Exactive hybrid quadrupole-Orbitrap mass spectrometer (Thermo Fisher Scientific) equipped with a nano ESI source. The protein identification was performed using PEAKS XPro (PEAKS Studio 10.6 build 20201015; Bioinformatics Solutions Inc. Waterloo, Ontario, CA). The analytical parameters were set as follows: search engine, sequest HT; protein database, Swissprot (Homo Sapiens); enzyme name, trypsin; parent mass tolerance, 10.0 ppm; false discovery rate <0.01; unique peptides ≥ 2. Proteins belonging to keratin were excluded from the identification results as contaminants. Based on triplicate experiments, we targeted the protein of average mass, ∼28 kDa, which was reproducibly identified in the PA-liposome-binding fraction.

### Reverse transcription PCR, protein expression, and purification of HSP27

AKI cells were washed twice with phosphate-buffered saline (pH 7.4) and collected by centrifugation (500*g*, 4 °C, 3 min), and total RNA was isolated as previously described (80). cDNA was generated using Transcriptor reverse transcriptase (Roche Diagnostics, Mannheim, Germany).

Human HSP27 cDNA (Accession number: AB020027) was amplified using the primers 5′-GGTGGTGGATCCATGACCGAGCGCCGC-3′ (forward) and 5′-GGTGGTCTCGAGTTACTTGGCGGCAGTCTC-3′ (reverse) from prepared cDNA (human AKI cells) by PCR, ligated with pET-28a vector (Novagen–Merck, Darmstadt, Germany), which carries an N-terminal 6 ×His tag, and transfected into Rosetta 2 (DE3) *E. coli* cells (Novagen). The expression and purification of the 6× His-tagged HSP27 protein using nickel-nitrilotriacetic acid agarose (Qiagen, Hilden, Germany) were performed as previously described (81).

### Western blotting

Western blotting was conducted as previously described (23). AKI and COS-7 cells were homogenized in ice-cold HEPES buffer (25 mM HEPES, pH 7.4, 100 mM NaCl, and 1 mM dithiothreitol). A protein-transferred polyvinylidene fluoride membrane (Pall Corporation, Port Washington, NY) was incubated with anti-HSP27 antibody (ab2790, Abcam, Cambridge, UK) and an anti-6× His antibody (D291-3S, Medical & Biological Laboratories, Nagoya, Japan).

### Lipid overlay assay

One hundred picomoles of various lipids was spotted in a nitrocellulose membrane (Lipid Strips; Echelon Biosciences). The membranes were blocked with 2% skim milk in phosphate-buffered saline (pH 7.4) for 1 h at 4 °C. After blocking, 10 ml of 3% fatty acid–free bovine serum albumin and 0.1% Tween 20 in phosphate-buffered saline (pH 7.4) containing 6× His-tagged HSP27 (final concentration: 20 nM) was added to the membranes. The membrane was incubated for 20 min at 4 °C and was then incubated with an anti-6× His antibody for 1 h at 4 °C, followed by incubation with anti-mouse IgG conjugated with horseradish peroxidase (Bethyl Laboratories, Montgomery, TX, USA) antibody. Finally, lipid-bound 6× His-HSP27 was visualized using an enhanced chemiluminescence Western blotting detection system (GE Healthcare, Little Chalfont, UK).

### Liposome-binding assay

The purified 6× His-tagged HSP27 protein (final concentration: 0.5 μM) was dissolved in HEPES buffer and incubated with the PA-containing or control liposomes at 4 °C for 30 min. Samples were ultracentrifuged at 200,000*g* at 4 °C for 1 h. The precipitate was dissolved in HEPES buffer containing 25 mM HEPES (pH 7.4), 100 mM NaCl, and 1 mM dithiothreitol. SDS-PAGE (15% acrylamide) was conducted, and separated proteins were stained with Coomassie Brilliant Blue or detected by Western blotting using an anti-6× His antibody.

To measure the binding activity between PA-containing liposomes and endogenous HSP27, AKI cells were washed two times with phosphate-buffered saline and lysed in HEPES buffer containing 25 mM HEPES (pH 7.4), 100 mM NaCl, and 1 mM dithiothreitol by sonication. After sonication, insoluble materials were removed by ultracentrifugation (200,000*g* for 30 min at 4 °C). The cell lysates were incubated with the PA-containing or control liposomes at 4 °C for 30 min. Samples were ultracentrifuged at 200,000*g* for 1 h at 4 °C. The precipitate was dissolved in HEPES buffer. SDS-PAGE (15% acrylamide) was conducted, and separated proteins were detected by Western blotting using an anti-HSP27 antibody.

### Plasmid constructs for COS-7 cell transfection

pEGFP-DGKα-Δ1–196, which is a constitutively active mutant (DGKα-CA) (29), and pEGFP-DGKα-CA-KD, a kinase-dead inactive mutant of DGKα-Δ1–196 in which Gly-435 is substituted with Asp (43), were generated previously. pmCherry-HSP27 was constructed by inserting a PCR fragment encoding HSP27 amplified from pET-28a-HSP27 into the EcoRI/SalI sites of the pmCherry-C1 vector (Sigma-Aldrich, St Louis, MO, USA).

### Confocal laser scanning microscopy

COS-7 cells seeded on coverslips were transiently transfected with plasmids using PolyFect reagent (Qiagen) as described by the manufacturer. After 20 h of transfection, the cells were incubated with the DGKα selective inhibitor CU-3 (29) (or DMSO alone as a control) in DMEM (final concentration: 10 μM) for 30 min to inhibit PA generation by DGKα-CA. The cells were then fixed in 4% paraformaldehyde. The coverslips were mounted with Vectashield (Vector Laboratories, Burlingame, CA, USA). Fluorescence images were obtained with an Olympus FV1000-D (IX81) confocal laser scanning microscope (Olympus, Tokyo, Japan) equipped with a UPLSAPO 60 × 1.35 NA oil at room temperature. EGFP fluorescence was excited at 488 nm, and mCherry fluorescence was excited at 543 nm. Images were obtained using FV-10 ASW software (Olympus).

### Blue native polyacrylamide gel electrophoresis

The purified 6× His-tagged HSP27 protein (final concentration: 2.3 μM) was dissolved in HEPES buffer and incubated with the PA-containing or control liposomes at 4 °C for 30 min. BN-PAGE (4% stacking gel and 6% separation gel) was carried out as previously described (82). The gel was incubated in denature buffer A (20 mM Tris-HCl (pH 7.4), 150 mM glycine, and 0.1% SDS) for 10 min at room temperature. After transfer, polyvinylidene fluoride membranes were washed with methanol, followed by incubation in denature buffer B (50 mM Tris-HCl (pH 7.4), 2% SDS, 0.8% β-mercaptoethanol) for 30 min at 50 °C. The proteins were detected using an anti-6× His antibody.

### Densitometry

Band intensities were quantified by densitometry using ImageJ software (https://imagej.nih.gov/ij/index.html) as described (https://lukemiller.org/index.php/2010/11/analyzing-gels-and-western-blots-with-image-j/)

### Statistical analysis

Data are represented as the means ± SD and were analyzed using one-way analysis of variance followed by Tukey's or Dunnett's post hoc test for multiple comparisons or two-tailed *t* test for the comparison of two groups using Prism 8 (GraphPad Software, San Diego, CA, USA) to determine any significant differences. *p* < 0.05 was considered significant.

## Data availability

The data that support the findings of this study are available from the corresponding author [sakane@faculty.chiba-u.jp] upon reasonable request.

## Conflict of interest

The authors declare that they have no conflicts of interest with the contents of this article.
